# Endocrine and paracrine characteristics of neuroendocrine prostate cancer

**DOI:** 10.3389/fendo.2022.1012005

**Published:** 2022-11-11

**Authors:** Tarana Arman, Peter S. Nelson

**Affiliations:** ^1^ Division of Human Biology, Fred Hutchinson Cancer Center, Seattle, WA, United States; ^2^ Division of Clinical Research, Fred Hutchinson Cancer Center, Seattle, WA, United States

**Keywords:** prostate cancer, secretion, paracrine, endocrine, neuroendocrine

## Abstract

Prostate cancer is a common malignancy affecting men worldwide. While the vast majority of newly diagnosed prostate cancers are categorized as adenocarcinomas, a spectrum of uncommon tumor types occur including those with small cell and neuroendocrine cell features. Benign neuroendocrine cells exist in the normal prostate microenvironment, and these cells may give rise to primary neuroendocrine carcinomas. However, the more common development of neuroendocrine prostate cancer is observed after therapeutics designed to repress the signaling program regulated by the androgen receptor which is active in the majority of localized and metastatic adenocarcinomas. Neuroendocrine tumors are identified through immunohistochemical staining for common markers including chromogranin A/B, synaptophysin and neuron specific enolase (NSE). These markers are also common to neuroendocrine tumors that arise in other tissues and organs such as the gastrointestinal tract, pancreas, lung and skin. Notably, neuroendocrine prostate cancer shares biochemical features with nerve cells, particularly functions involving the secretion of a variety of peptides and proteins. These secreted factors have the potential to exert local paracrine effects, and distant endocrine effects that may modulate tumor progression, invasion, and resistance to therapy. This review discusses the spectrum of factors derived from neuroendocrine prostate cancers and their potential to influence the pathophysiology of localized and metastatic prostate cancer.

## Introduction

Prostate cancer (PC) is the second most commonly diagnosed cancer worldwide among men (1). The American Cancer Society has estimated that ~270,000 men will be diagnosed with PC in 2022 in the United States and PC will cause in excess of 34,000 deaths (2). The vast majority of men diagnosed with PC present with localized disease and the histology of these tumors are primarily adenocarcinomas with distinctive architectures codified as Gleason patterns (3). The most characteristic feature displayed by the vast majority of localized and metastatic PCs involves the expression of the androgen receptor (AR) and a program of genes/proteins regulated by the AR including a group of secreted factors such as prostate specific antigen (PSA) (4). In addition to specifying prostate epithelial lineage and regulating metabolic and secretory functions, the AR serves as a key therapeutic target both for localized tumors and metastases (5, 6).

While the vast majority of PCs are adenocarcinomas with secretory epithelial features and an active AR program, PCs with a spectrum of other histological characteristics also occur. Among these are PCs with qualities of neuroendocrine (NE) cells (7). These prostate neuroendocrine carcinomas (NEPCs), representing <1% of all localized PCs, exhibit features found in benign NE cells and in NE carcinomas arising in other organs and tissues (8, 9). In the context of localized NEPC, the origin of these tumors has not been conclusively established as they may arise from resident benign NE cells or from stem-like, basal or luminal cells that usually serve as the cell of origin for typical adenocarcinoma (10–12). In the setting of metastatic PC (mPC), tumors with NE features are more common, ranging from 10-30% depending on the markers used for classification and the disease state with respect to the application of therapeutics that suppress AR signaling (13, 14). Preclinical models have demonstrated the occurrence of transdifferentiation whereby tumor cells with a typical epithelial phenotype and active AR program lose AR activity and gain NE characteristics during the development of resistance to AR repression (15–17). With the advent of more potent AR signaling inhibitors (ARSI) such as abiraterone and enzalutamide, the frequency of tumors with NE phenotypes is increasing (18). One feature of metastatic NEPC is the downregulation or complete absence of AR expression and AR signaling (18, 19). Notably, since a subset of these tumors harbor underlying genomic alterations commonly observed in AR-active PCs that serve to promote AR oncogenic functions such as TMPRSS2-ERG rearrangements and structural alterations in the AR locus – it is likely that these metastatic PCs arise through transdifferentiation processes that are usually repressed by an active AR program, and enhanced by the loss of key tumor suppressors that influence cell reprogramming such as *TP53* and *RB1* (13, 19–25).

While pure NEPC is evident in some tumor biopsies including a subset with small cell histology that is indistinguishable from small cell carcinomas arising in other organs such as the lung, other tumors show mixtures of ARPC and NEPC cells indicating a degree of intratumor heterogeneity (14, 26). Currently, neuroendocrine small cell carcinomas are primarily characterized by morphological features, lack of AR expression, and a higher expression of several canonical markers that reflects NE cell differentiation, e.g. the transcriptional factors (TFs) ASCL1, NEUROD1, INSM1, and NE function, for example, the secreted proteins synaptophysin (SYP), chromogranin A (CgA) and neuron specific enolase (NSE) (14, 27, 28).

The role of the TFs in NE differentiation has been an active area of investigation. ASCL1 plays a key role in promoting and maintaining NE features of luminal cell types by modulating chromatin dynamics, supporting lineage plasticity, and directly regulating the expression of secreted NE proteins (29). Similarly, NEUROD1 has been studied in the context of several aggressive neural/neuroendocrine carcinomas and are important for their survival, invasion, and metastasis (30). INSM1 is a zinc-finger transcriptional factor that functions as a transcriptional repressor, thus regulating cell cycle arrest and facilitating NE differentiation (31). On the other hand, endocrine and paracrine functions of NE secretory proteins, despite being some of the most commonly used NE markers have not been established. Most of these canonical markers are not specific to the prostate, but are rather expressed in a variety of tumors that belong to the diffuse neuroendocrine system (32).

## Characteristics of neuroendocrine cells in the normal prostate and prostate carcinoma

**Figure 1:**
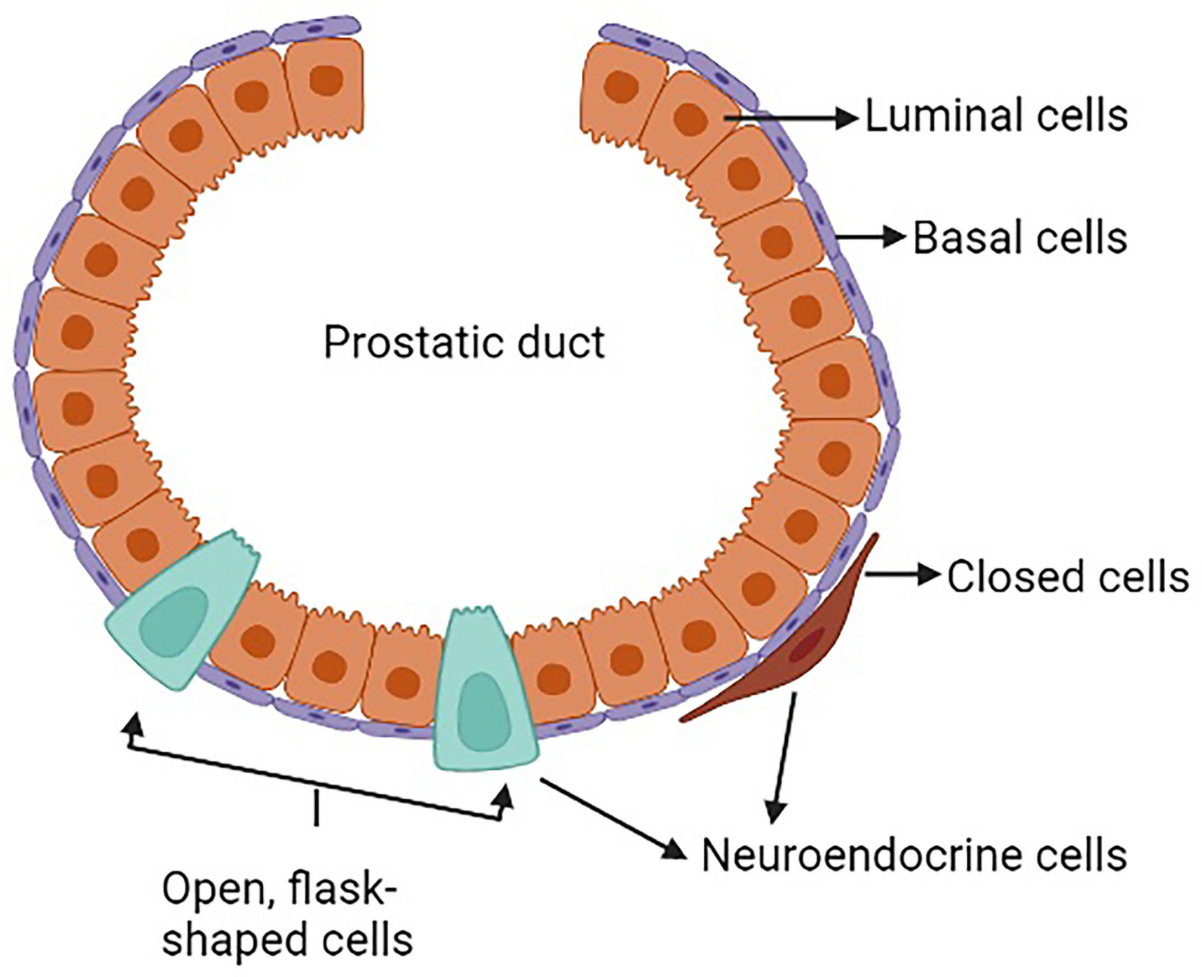
Neuroendocrine cells in a normal prostate.

The prostate is a complex secretory organ comprised of multiple cell types broadly partitioned into epithelium and stroma. The stroma includes predominant resident cell types of smooth muscle, fibroblasts, vascular endothelium and nerves, which are variably infiltrated with transitory inflammatory cell populations that include neutrophils, lymphocytes, and macrophages (33–35). The epithelial compartment is comprised of two primary cell types: basal and luminal/secretory cells, and a minor (<1%) population of neuroendocrine (NE) cells (7, 10, 12, 36) (**Table 1**). Increasingly sophisticated molecular profiling studies now subdivide these broadly classified types into subtypes with distinctive functions such as those with stem cell/self-renewal capabilities (35, 46, 47). The rare resident NE cells are not easily appreciated using standard H&E staining. They are better identified through immunohistochemistry (IHC) using common markers including chromogranin A (CgA), Synaptophysin (SYP), neuron-specific enolase (NSE), neural cell adhesion molecule (NCAM), Forkhead-box A2 (FOXA2) and CXC chemokine receptor 2 (CXCR2) (12, 48). NE cells are androgen-insensitive and postmitotic and have been shown to be preferentially situated around Ki-67 positive epithelial cells (49, 50), which are highly proliferative. Morphologically, there are two distinct populations of NE cells in the prostate: open, flask-shaped cells with long and slender extensions reaching the lumen, and closed cells without luminal extensions (51) (**Figure 1**). Although the distinct roles of these two NE sub-populations have not been addressed specifically, there is a distinction in their ability to interact with the prostate environment. Closed cells can only receive basal stimuli, whereas, open cells can also receive luminal stimuli (52). The functional role of NE cells in the mature prostate is not well-defined. Electron microscopic studies have shown that NE cells secrete a variety of products, including serotonin, histamine, chromogranin A and other related peptides, calcitonin, calcitonin gene-related peptide, katacalcin, neuropeptide Y, vasoactive intestinal peptide (VIP), bombesin/gastrin releasing peptide (GRP), somatostatin, alpha-human chorionic gonadotropin (aHCG), parathyroid hormone-related protein (PTHrP), thyroid stimulating hormone-like peptide, cholecystokinin, adrenomedullin and vascular endothelial growth factor (VEGF) (8, 53). The potential role of these factors on prostate cancer pathobiology has been detailed in **Table 2**, however, in normal physiology these secreted factors have growth promoting and angiogenic properties, justifying the proximity of NE cells near to the highly proliferative cells. The receptors for some of these products are detected in benign and neoplastic prostate epithelium. This suggests, the possible roles of NE cells in the regulation of growth and differentiation of the developing prostate and also regulation of secretory processes in the mature gland (8, 51, 53). Another postulated role of NE cells is regulation of sperm function, because many of the aforementioned secretory products are also detected in the seminal fluid (11, 53).

**Table 1:** Common cell types comprising the normal prostate.

Cell type	Markers for identification	Function	References
Smooth Muscle Cells	• ACTA2, MYH11, MT1A, RGS5. • Identified by a combination of morphology, tissue position and lack of markers for epithelial cells, endothelial cells and leukocytes. • Vimentin and platelet-derived growth factors are sometimes used as markers, but their expression not restricted to fibroblasts.	• Contractile function altering prostate glandular shape for urination vs ejaculation. • Structural component of the stroma.	(37, 38)
Fibroblast Cells	• APOD, FBLN1, PTGDS, DCN • Vimentin	• Production of extracellular matrix • Structural component of stroma • Production of signaling molecules that influence epithelial development and function	(35)
Vascular Endothelial Cells	• VEGF • CD34 • CD31 • CD200	• Growth and maintenance of differentiation of prostatic epithelium.	(39, 40)
Nerves and Nerve Cells	• Protein S100 • VAChT • Tyrosine hydroxylase (TH)	• Receives sympathetic input *via* hypogastric nerves. • Receives parasympathetic input *via* pelvic nerve. • These nerves also provide sensory inputs to the gland.	(41)
Neutrophils	• CD16 • CD45	• Role in tumor development by producing cytokines, proteases, and reactive oxygen species (ROS) and interacting with other immune cells	(42)
Lymphocytes	• CD3 • CD4 • CD19	• Critical components of antitumor immunity. • Aid in antibody production.	(42)
Macrophages	• iNOS • CD38 • Ym1 • CD206	• Promotes cell proliferation of normal prostate epithelial cells	(43)
Basal Epithelial Cells	• Cytokeratins 5 and 14 • p63 • CD49f, CD104, CD271	• Critical role in maintaining ductal integrity. • Maintenance of survival of luminal cells.	(44)
Luminal/Secretory Epithelial Cells	• Cytokeratins 8 and 18 • CD26, CD38 • KLK3 • Androgen receptor (AR) • Prostate specific antigen (PSA)	• Prostate development. • Androgen mediated regeneration.	(45)
Neuroendocrine Cells	• CgA • SYP • NSE • NCAM/CD56) • FOXA2 • CXCR2	• Function not well studied. • Interacts with nearby epithelial cells in a paracrine manner.	(12)

**Table 2:** Secreted factors from neuroendocrine cells and potential roles in PC pathobiology.

Symbol	Name	Role	Reference
CgA	Chromogranin A	• Marker for NED • Elevated serum level is associated with poor prognosis and is inversely correlated with overall survival in men with CRPC.	(54, 55)
CgB	Chromogranin B	• Marker for NED • Involved in PC transdifferentiation	(56, 57)
SYP	Synaptophysin	• Marker for NED • Detected in circulating tumor cells of CRPC patients and the expression levels directly correlates with abiraterone and enzalutamide resistance.	(58)
ENO2	Enolase 2	• Isoenzyme of the glycolytic enzyme enolase. • Marker of NEPC; upregulated as a result of IL8 mediated downregulation of FOXA1	(59, 60)
SCGN	Secretagogin	• Neuroendocrine marker • Correlates with an increased risk of disease relapse after radical prostatectomy	(61, 62)
NPY	Neuropeptide Y	• Growth promoting factor in various malignancies. • Key regulator of energy metabolism in PC cells. • NPY neural axis regulates cancer cell survival, metabolism, and therapy resistance.	(63)
CGRP	Calcitonin-gene related peptide	• Increase invasiveness and promoting tumor growth in bone microenvironment.	(64, 65)
CT	Calcitonin	• Elevated in advanced prostate cancer along with calcitonin receptor (CTR). • CT-CTR axis promotes PC cell growth, invasion and epithelial-to-mesenchymal transition (EMT).	(8) (66, 67)
NTS	Neurotensin	• Expressed in LnCaP cells as a response to androgen-withdrawal. • Induces tumor cell transdifferentiation to NE-like cells through (NTSR1/3) signaling. • NTSR1 also has a frequently elevated expression in metastatic lymph nodes	(68)
AM	Adrenomedullin	• Expressed in LnCaP cells as a response to androgen withdrawal and maintains a NE phenotype. • Supports hormone independent tumor growth and neovascularization by supplying/amplifying signals for neoangiogenesis and lymphangiogenesis.	(69) (70)
IL8	Interleukin 8	• Expressed in both benign and malignant NE cells. • Increased expression associated with reduced FOXA1 expression in NEPC cells. • Paracrine and autocrine effects: cell survival and proliferation; NED.	(71) (72, 73)
GRP	Gastrin releasing peptide (mammalian homologue of bombesin)	• Increased expression as a response to androgen withdrawal, activates the GRP/GRP-receptor (GRP-R) pathway, in turn activating the NF-κB and increased levels of AR-splice variant (AR-V7). • GRPR expression also amplifies in CRPC.	(74) (75)
SS	Somatostatin	• Inhibits cytokine release from immune cells. • Somatostatin receptor scintigraphy (SRS) can identify NE features in prostate cancer and identify metastatic lesions.	(76–79)
VIP	Vasoactive intestinal peptide	• Increases expression of VEGF. • Induces NE differentiation in LnCaP cells through PKA, ERK1/2 and PI3K signaling.	(80, 81)
VEGF	Vascular endothelial growth factor	• Increased expression associated with a more aggressive phenotype by aiding in neovascularization of carcinomas. • Increased metastasis to lymph nodes	(82, 83)
PTHrP	Parathyroid hormone related protein	• Enhances proliferation of LnCaP cells at low levels of androgen, by stabilizing the AR protein through tyrosine phosphorylation and preventing ubiquitination. • Induces epithelial-to-mesenchymal transition (EMT) in prostate cancer cells along with promoting invasion, tumorigenicity and metastasis. • Protection of neighboring prostate cancer cells from Docetaxel (Doc) induced apoptosis. • Positively regulates bone marrow microenvironment, increasing the angiogenic potential and tumor growth.	(84) (85–87)
HCG	Human chorionic gonadotropin	• Associated with poor prognosis in PC patients. • Promotes cell migration and invasion *via* promoting ERK1/2 phosphorylation and MMP-2 upregulation in DU145 cells.	(88, 89)
5-HT	Serotonin	• Cell growth factor for PC cells. • Promotes dedifferentiation of LnCaP cells by maintaining an increased level of cAMP. • Activates MAPK/Erk and PI3K/Akt pathways to induce proliferation, migration, and differentiation.	(90, 91)
CCK	Cholecystokinin	• Induced by cysteine protease cathepsin B (CTSB) • Supports self-renewal of PC stem cells (CSCs)	(92)

## Neuroendocrine prostate cancer and neuroendocrine transdifferentiation

Detailed mechanisms influencing prostate carcinogenesis and tumor development have been previously reviewed (93, 94). Briefly, invasive prostate adenocarcinoma may develop directly from differentiated secretory epithelium, epithelium with stem-like characteristics, or from precursor lesions such as high grade prostatic intraepithelial neoplasia (HGPIN). Prostate adenocarcinoma exhibits characteristic features that include cytologic atypia with enlargement of nuclei and nucleoli, loss of the basal cell layer, branching morphogenesis, and ultimately loss of gland formation (93–95). While localized PC is generally treated by surgical removal or radiation therapy, metastatic PC requires systemic therapies – primarily drugs that repress AR activity. While most PCs resist AR targeting by maintaining or amplifying AR signaling, a subset of PC cells is capable of transdifferentiation – a process whereby a differentiated AR-active tumor cell with secretory luminal cell characteristics, change phenotypes with the resultant loss of AR/luminal cell features and the gain of NE attributes that may include alterations in morphology as well as the expression of NE transcription factors and secretory proteins indicative of differentiated NE cell types (25). This process termed neuroendocrine differentiation (NED) is an adaptive mechanism of PC cells to achieve therapy resistance as AR signaling is no longer operative or required for cell survival and proliferation (28). Determining the cellular mechanisms that initiate NED remains an active area of investigation, although studies have shown that loss of tumor suppressor proteins such as *PTEN*, *RB1*, *TP53* increases the chances of tumors to acquire neuroendocrine like features (96, 97). However, these tumor suppressors appear to function as permissive rather than deterministic factors. While loss of AR activity and attendant enforcement of epithelial lineage is a key feature contributing to NED, the precise molecular switches responsible for gaining NE functions remain to be identified.

While a complete transition from ARPC to NEPC has been shown to occur in patients, detailed autopsy studies have shown that metastatic tumors may comprise heterogenous populations of ARPC and NEPC that co-exist. Inter- and intra-tumor heterogeneity with respect to tumor cells with ARPC and NEPC phenotypes is well-documented. Notably, other than representing a clear mechanism/pathway for bypassing AR-directed treatment, the role and influence of NE cells in PC pathology, particularly with respect to tumor progression and therapy resistance is not completely understood. This is relevant in view of the potential for NE-associated paracrine and endocrine factors to influence the behavior of non-NE cell types – either locally or distantly. In this context, a previous study reported that the NE cells promoted the growth of castration sensitive LnCaP cells, when grown as a xenograft in castrated mice (98). Further, NE cells were also shown to enhance the migration and metastasis of ARPC cells in the presence of androgen (99). Thus, NEPC cells may promote the continued survival of ARPC cells in an androgen deprived environment possibly through paracrine and endocrine mechanisms (discussed later).

## Neuroendocrine carcinomas in non-prostate organs and tissues

Neuroendocrine tumors (NETs) are generally classified as neoplasms with both neural- and endocrine-like characteristics, and these malignancies often have the ability to store and secrete different peptides and neuroamines (100). Although rare, NETs can occur anywhere in the body. Some of the common sites of NET occurrence are the GI tract, lungs and pancreas. The definition of NE cells has changed over the years and in many instances their origins are still not clear. The generally accepted criteria for defining NE cells are: (1) production of a neurotransmitter, neuromodulator or neuropeptide hormone, (2) the presence of dense -core secretory granules from which hormones are released by exocytosis, and (3) the absence of axons and synapses (101). This section briefly discusses several of the more common types of NETs, though as noted above, NETs can arise in nearly every organ/tissue in the body and not all are described here, for example neuroblastoma which is a NE tumor type arising almost exclusively in children (102).

### Gastric neuroendocrine tumor

These neoplasms are derived from enterochromaffin-like cells (ECL cells) of the gastric mucosa (103). Over the last several years, the incidence of gNETs is increasing, partly due to improved diagnostic techniques (104). gNETs can be clinically functioning (symptomatic) or silent (non-symptomatic) (105). Based on clinicopathological characteristics, and therapeutic and prognostic implications, gNETs are further subdivided into four types (Type I-Type IV) (104): Type I gNET comprise 70-80% of gNETs and are associated with autoimmune chronic atrophic gastritis (103, 106). These patients often suffer from hypergastrinemia (increased gastrin production by G cells) (103). Patients with type I tumors are usually asymptomatic, and the tumors are rarely metastatic (<2%) (106). However, these tumor cells strongly stain positive for NE markers: chromogranin A (CgA) and neuron specific enolase (NSE) (106), but have very low proliferation rates; Type II gNET represent~7% of gNETs and behave like type I tumors and are caused by gastrinomas. These tumors show an increased staining for CgA compared to the type I tumors and exhibit a higher metastatic potential (103); Type III gNET are aggressive with tissue invasion and metastasis and have a poor prognosis (106). The tumor cells also show a greater frequency of staining for the proliferation marker Ki-67, but are negative for CgA (106); Type IV gNET are very rare, but are highly malignant and exhibit very high Ki67 staining. The tumor cells may lack CgA expression but stain positive for other NE markers such as synaptophysin (SYP) and NSE (106).

### Pancreatic neuroendocrine tumors

These are rare neoplasms that represent 1-2% of all pancreatic tumors (107). They were originally thought to arise from the islets of Langerhans, however, evidence suggest an origin from the pluripotent stem cells in the pancreatic ductal/acinar system (108). pNETs produce a range of hormones, including insulin, glucagon, somatostatin, and vasoactive intestinal peptide (VIP) (107). Although most pNETs occur sporadically, about 10% are associated with underlying genetic syndromes including multiple endocrine neoplasia type I (MEN1), type IV (MEN4), von Hippel-Lindau disease (VHL), neurofibromatosis type I (NF1), or tuberous sclerosis complex (TSC) (109, 110). Like the gNETs, pNETs are also classified into functional and non-functional tumors. Functional tumors elicit systemic symptoms through excessive secretion of hormones.

### Lung neuroendocrine tumors

These are a heterogenous family of neoplasms in the lung, that arises from the Kulchitzky cells of the bronchial mucosa (111). They are classified into four distinct histologic variants, namely, typical carcinoid (TC), atypical carcinoid (AC), large cell neuroendocrine carcinoma (LCNEC) and small cell lung carcinoma (SCLC) (112).

SCLC is the most aggressive form of lung cancer. SCLC was originally thought to arise *de novo* from resident neuroendocrine lung cells, but recent evidence from model systems suggests alternative cells of origin such as alveolar type 2 cells (113–115). Similar to NEPC, SCLC can also emerge following targeted therapy for lung adenocarcinoma. For example, resistance to epidermal growth factor receptor (EGFR) inhibitors can result through tumor cell transdifferentiation to SCLC phenotypes which no longer depend on EGFR signaling (116). SCLC is well known to produce a variety of paraneoplastic syndromes that result from the production of hormones such as adrenocorticotrophic hormone (ACTH) (117).

Notably, SCLC shares a strong similarity of chromatin structure and gene expression with NEPC (118). A detailed study of various small cell neuroendocrine cancers (SCNCs) across multiple tissues, shows that these cancers share a convergence of molecular signatures (119, 120). Further, as tumor cells progress towards a small cell neuroendocrine (SCN) phenotype through transdifferentiation, they become increasingly independent of tissue of origin and cluster with SCNCs derived from different tissue types (120). Progress in the clinical treatment of SCNCs has been very slow and improving the outcomes of these aggressive tumors by exploiting the mechanisms underlying their genesis has yet to be fully realized.

### Merkel cell carcinoma

Merkel cells are highly specialized cells located in the epidermis that function as pressure receptors and may originate from neural crest cells or from epidermal progenitors (121). While originally considered to be the cell of origin of MCC, which is also classified as primary neuroendocrine carcinoma of the skin, primary small cell carcinoma of the skin and trabecular carcinoma of the skin, more recent studies indicate that these tumors arise from a Merkel cell precursor or from resident fibroblasts *via* transdifferentiation (121, 122). Drivers of MCC include the Merkel cell polyoma virus – accounting for ~80% of MCCs, and the combination of *TP53* and *RB1* loss, which occur in the remainder (123–126). MCCs share many features with NE carcinomas arising in other tissues including the expression of SYP and CGA and the neural transcription factor NEUROD1 (27).

In summary NETs are a diverse group of neoplasms, distinguished by site of origin, degree of aggressiveness and function. Although site-specific, most of the NETs express immunohistochemical markers like CGA and SYP. These tumors also produce a similar range of bioactive compounds or hormones (127) that may influence tumor cells at distant sites or produce pathological host effects that are collectively termed as paraneoplastic syndrome (PNS). A PNS may be endocrine – resulting from a specific hormone produced by the cancer, or immune mediated. Though PNS are rare when considering all human cancers, they occur more frequently in NETs. Several well-characterized PNS results from the secretion of excess hormones such as ACTH and others that produce neurologic alterations due to the production of autoantibodies (117). For most of these NETs, surgery and chemotherapy remain the primary curative option if the cancer is identified while localized to the primary site. This is, however, not feasible in NEPC as most patients do not present with localized, organ-confined disease.

## The endocrine and paracrine characteristics of NEPC

**Figure 2:**
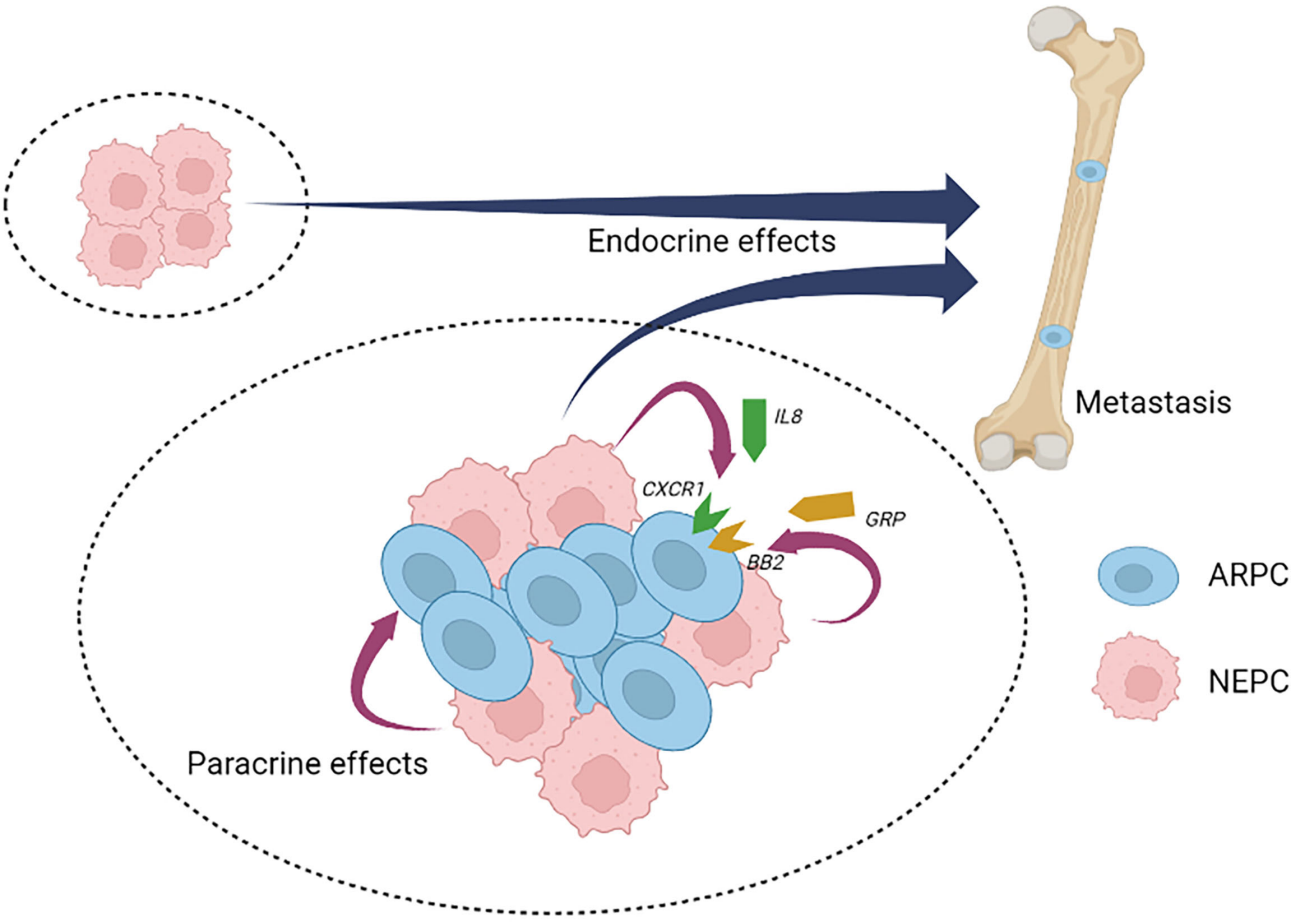
Neuroendocrine prostate cancer paracrine and endocrine signaling. Neuroendocrine prostate cancer (NEPC) cells produce and secrete a spectrum of peptides and proteins with paracrine effects that influence local cell types and endocrine effects that influence distant cell types, tissues and organs. NEPC-derived factors have the potential to promote the progression and therapy resistance of non-NEPC ARPC cells within heterogenous tumors (intra-tumor heterogeneity) or in situations where inter-tumor heterogeneity occurs. NEPC, neuroendocrine prostate cancer; ARPC, AR active prostate adenocarcinoma; IL8, interleukin 8; CXCR1, the IL8 receptor C-X-C motif chemokine receptor 1; GRP, gastrin releasing peptide; BB2, the GRP/Bombesin receptor 2.

Analogous to benign NE cells found in the normal prostate, NEPC cells are also capable of secreting a wide range of neuropeptides and other factors (11, 12, 51). To date, studies defining the role(s) of NEPC derived secreted products with respect to PC pathogenesis and response to treatment are limited, but the published reports indicate the importance of further work in this field. Collectively, more than 20 distinct NEPC-derived secreted factors have been identified that are capable of exerting effects on PC adenocarcinoma (**Table 2**). Recently, it was discovered that neuropeptide Y has a paracrine effect on PC cells by influencing apoptosis, motility, and resistance to radiation therapy (63). The cytokine Interleukin 8 (IL-8) is produced by NEPC cells and is capable of activating non-NE PC cells *via* the IL-8 receptor CXCR1 with downstream signaling that is capable of driving androgen-independent proliferation and tumor cell invasion (71) (**Figure 2**). Adrenomedullin, a multifunctioning peptide, is produced by PC cells after androgen depletion, and exerts autocrine signaling that induces a NE-like transdifferentiation phenotype switch (69). The neuropeptide Bombesin/Gastrin Releasing Peptide (GRP) which is expressed in NEPC, exerts mitogenic effects toward PC cells *via* bombesin receptor (BB2) signaling and may also contribute to androgen-independent growth (74) (**Figure 2**).

The NEPC disease state is often associated with metastatic dissemination. Caveolin-1 is an oncogenic membrane protein associated with extracellular matrix organization, cell migration and signaling (128). In prostate cancer cells, caveolin-1was shown to exert paracrine effects that increase PC proliferative activity aiding in perineural invasion and reduced apoptosis (129). The presence of caveolin-1 in tumor derived exosomes also has a paracrine effect on PC cells, driving the induction of cancer stem cell phenotypes, epithelial- mesenchymal transition, and neuroendocrine differentiation (130). As discussed previously, NEPC cells have been shown to maintain ARPC adenocarcinoma tumor growth after castration, by releasing uncharacterized factors that act to increase AR expression and activity *via* paracrine and endocrine signaling (98). NEPC cells have also been shown to promote the development of adenocarcinoma pulmonary metastasis (99). Gelsolin is a multifunctional actin-binding protein (131), that shows an increased expression as a response to extracellular factors produced by NEPC cells. Gelsolin overexpression promotes epithelial cell invasion and an increase in cell migration (99).

As mentioned before, NEPC cells can also exert systemic effects through PNS. Though overall extremely rare, a range of PNSs have been shown to arise in the context of aggressive and metastatic PC, and notably NEPC [reviewed by Hong et al. (132)]. Though unusual, the following paraneoplastic syndromes have been attributed to PC: (1) The syndrome of inappropriate antidiuretic hormone secretion (SIADH) is a cause of hyponatremia (133). Patients with SIADH have an elevated antidiuretic hormone (ADH) level in the serum, which then acts on the distal tubules and collecting ducts of the nephron and in turn increase water resorption (132). Although SIADH is very rare in PC patients, there have been a few clinical cases reported (133), and PC tumor cells have been shown to express ADH (134). (2) Cushing’s syndrome is caused by an excess of circulating serum cortisol as a result of excess adrenocorticotrophic hormone (ACTH) (132). In PC, Cushing’s syndrome is primarily associated NE-differentiation to small cell carcinoma (135). (3) Humoral hypercalcemia, is caused by the inappropriate release of parathyroid hormone related peptide (PTHrP) by the tumor cells, which stimulates bone resorption throughout the skeletal system (132). Although very rare in typical PC, NEPC cells have been reported to synthesize and secrete PTHrP with both paracrine signaling effects toward ARPC and endocrine effects contributing to hypercalcemia (84, 136). Several other syndromes resulting from autoimmune responses have been reported in rare instances to be associated with PC and NEPC including Evan’s syndrome which involves immune-mediated hemolytic anemia and thrombocytopenia (137); exfoliative dermatitis (138); polymyalgia rheumatica (139); myasthenia gravis (140); dermatomyositis (141); paraneoplastic jaundice (142) and others (132).

## Role of nerves in the development of prostate cancer

**Figure 3:**
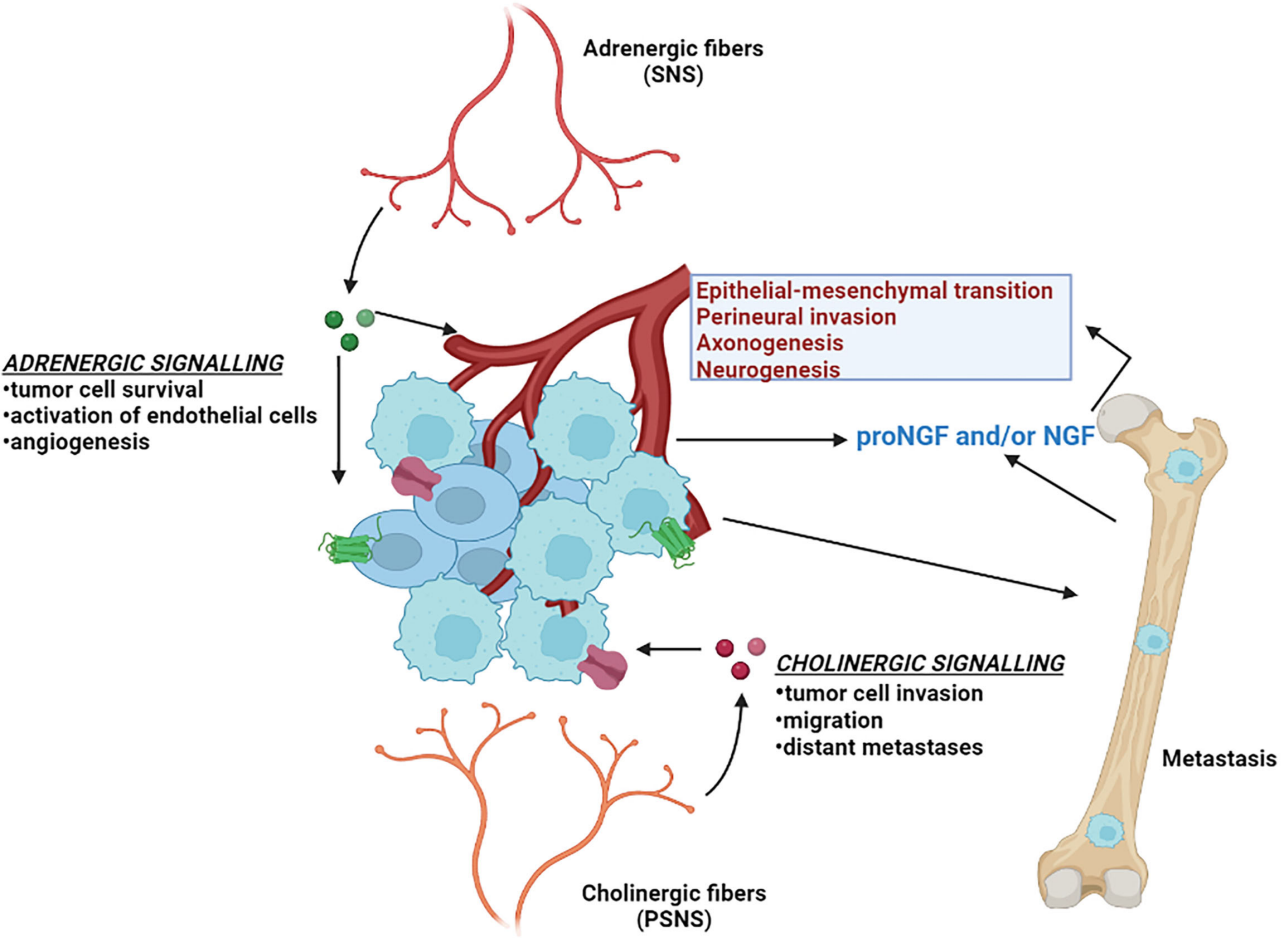
Neural signaling and prostate cancer. Different aspects of prostate cancer development and progression are supported by the autonomic nervous system. The adrenergic fibers of the sympathetic nervous system (SNS) release noradrenaline, that stimulate the beta-adrenergic receptors and supports angiogenesis and tumor cell survival. The cholinergic fibers of the parasympathetic nervous system (PSNS) secrete acetylcholine, that stimulates the cholinergic receptors and contributes to the pathogenesis of aggressive and malignant prostate cancer tumor variant. Invasive prostate cancer cells also secrete neurotrophic factors such as nerve growth factor (NGF) that further stimulates the growth of the autonomic nerve growth into the tumor microenvironment.

In addition to the potential for NE cells to exert effects on non-NE tumor cells *via* paracrine and/or endocrine effects, nerve cells have also been shown to influence tumor cell behaviors. This section will briefly summarize what is known regarding the role of neural signaling in prostate cancer progression (**Figure 3**). In addition to fibroblasts, endothelial cells and immune cells, neurons and nerve fibers are integral and functional components of tumor microenvironments (143). The processes of neurogenesis (increased numbers of neurons/nerves) and axonogenesis (tumor induced neural sprouting within or toward tumor microenvironments) can be driven by neurotrophic growth factors released by cancer cells and are emerging as hallmarks of aggressive cancer types (143, 144). The involvement of nerves in cancer has been studied in the context of perineural invasion (PNI), which is the process of neoplastic invasion of nerves contributing to metastatic spread (145, 146). However, until recently, nerves were generally considered passive components of cancers (144). Studies from multiple cancer models have now demonstrated the active involvement of the parasympathetic nervous system (PSNS) and sympathetic nervous system (SNS) in cancer progression and tumorigenesis (147) PSNS and SNS are components of the autonomic nervous system. SNS controls the “flight or fight” response and PSNS controls the “rest and digest” processes. Cholinergic fibers of the PSNS transmits impulses to other nerve cells or muscle fibers by transmitting acetylcholine. Adrenergic fibers of the SNS regulates the function of nearby and distant muscles and also components of the central nervous system by transmitting epinephrine or norepinephrine.

Several landmark studies regarding tumor innervation and its effect in PC have been reported in the last several years and have been reviewed in-depth (144, 148, 149). *In-vitro* co-culture experiments with dorsal root ganglia showed an increased proliferation of human PC cell lines (150), suggesting an interdependence of carcinoma cells and neurons in PNI contributing to PC progression. The importance of nerves in PC progression was further confirmed when surgical denervation showed inhibition of prostate tumor development in mouse models (151). The same group also parsed out the roles of the two distinct autonomic nerve types: (i) adrenergic fibers of the SNS in promoting tumor cell survival and establishing the initial stages of cancer development by acting through the β_2_ and β_3_-adrenergic receptors, and (ii) cholinergic fibers of PSNS in supporting tumor cell invasion, migration and distant metastases through stromal Chrm1 (cholinergic receptor muscarinic 1)-mediated signals (151). In alignment with these findings, clinical evidence has demonstrated that patients with spinal cord injuries resulting in functional denervation of the prostate have lower incidence rates of PC (152).

The mechanisms by which nerves influence the pathogenesis of solid tumors is beginning to be understood. Tumors rely on angiogenesis to expand beyond certain physiological constraints relating to oxygenation and the delivery and elimination of metabolites (153). Adrenergic nerve fibers release noradrenaline into the tumor microenvironment that stimulates β_2_-adrenergic receptor expression, resulting in the activation of endothelial cells and angiogenesis, which in turn supports PC growth (154). PCs of higher Gleason grade have been reported by exhibit greater innervations than PCs of lower grade or benign prostatic hyperplasia (144). Various neurotrophic growth factors produced by cancer cells can contribute to the increased axonogenesis in PC. Overexpression of the precursor of nerve growth factor (proNGF) has been reported in PC accompanied by increases in nerve density (155). Furthermore, it was shown that granulocyte colony-stimulating factor (G-CSF) supports the survival of sympathetic nerve fibers and promotes aberrant outgrowth of parasympathetic nerve fibers in PC models (156).

Several studies have evaluated the role of nerve growth factor (NGF) in the development of CRPC and NEPC disease states. *Tropomyosin receptor kinase A* (TrkA) receptors activated *via* nerve growth factor (NGF) signaling have been shown to mediate proliferation, invasiveness and epithelial-mesenchymal transition (EMT) in CRPC cells (157). In the context of ARPC treatment resistance, ADT has been shown to activate the transcription factor ZBTB46, which consequently regulates the activation of NGF. NGF in turn promotes NEPC differentiation by interacting with Chrm4 (cholinergic receptor muscarinic 4) (158). Another recent study reported that patients who subsequently developed metastatic CRPC had elevated adrenergic nerve fiber innervation in the primary prostate tumors. High levels of the neurotransmitter norepinephrine, which is produced by sympathetic nerves, was shown to induce NE-like alterations in PC cells, and these effects were effectively inhibited by β_2_-adrenenergic receptor blocker propranolol (159).

## Conclusions and future directions

Neuroendocrine prostate cancer, whether developing *de novo*, or through transdifferentiation, carries a very poor prognosis with rapid disease progression and very limited survival. As the frequency of metastatic NEPC appears to be increasing in the setting of more potent AR pathway blockade, new treatment approaches are needed. A notable feature of NE tumors involves their ability to exert effects toward other tumor cell types and benign host cells through endocrine and paracrine mechanisms. These secreted proteins provide a communication network between cancer cells and their adjacent microenvironment that may serve to drive tumor progression and treatment resistance. Preclinical studies have identified the potential therapeutic benefit of inhibiting the activity of the signaling pathways activated by these NE-derived molecules. However, the full repertoire of NEPC-derived secreted factors – the secretome - remains to be identified and characterized. A recent comprehensive secretome study of different subtypes of SCLC underscores the benefit of understanding the aspects of tumor biology that have extracellular influence (160). A thorough understanding of the NEPC secretome: individual factors and combinations - has the potential to widen our understanding of peptides/proteins that can act in an endocrine/paracrine manner to create tumor macro- and microenvironments conducive to tumor survival and growth. Characterizing the interactions between NEPC and ARPC cells also has the potential to identify key drivers of cancer progression and therapy resistance that could serve as effective targets for future drug development.

## Data availability statement

The original contributions presented in the study are included in the article/supplementary material. Further inquiries can be directed to the corresponding author.

## Author contributions

Writing and editing of the manuscript, TA and PN. All authors contributed to the article and approved the submitted version.

## Funding

We gratefully acknowledge support from the Pacific Northwest Prostate Cancer SPORE CA097186; NCI R01 CA234715, 1R01 CA266452, PC170350P1, the Prostate Cancer Foundation and the Institute for Prostate Cancer Research.

## Acknowledgments

We thank members of the Nelson laboratory, John Lee and Michael Haffner for helpful comments and suggestions.

## Conflict of interest

The authors declare that the research was conducted in the absence of any commercial or financial relationships that could be construed as a potential conflict of interest.

## Publisher’s note

All claims expressed in this article are solely those of the authors and do not necessarily represent those of their affiliated organizations, or those of the publisher, the editors and the reviewers. Any product that may be evaluated in this article, or claim that may be made by its manufacturer, is not guaranteed or endorsed by the publisher.
